# Canada Journal of Dental Science

**Published:** 1877-05

**Authors:** 


### CANADA JOURNAL OF DENTAL SCIENCE.

The publication of this journal, which has been suspended for some time, will be resumed on the first of July. At this we are much gratified; for it is an indication of a revival of professional interest in Canada; we would not intimate that there is less interest there than elsewhere, but it is encouraging to note signs of progress and vigor when and wherever it may be. We hope the Canada Journal will have a large subscription list from the United States. Dr. Geo. W. Beers, D. D. S., of Montreal is the editor.

